# Astrocytic Atrophy Following *Status Epilepticus* Parallels Reduced Ca^2+^ Activity and Impaired Synaptic Plasticity in the Rat Hippocampus

**DOI:** 10.3389/fnmol.2018.00215

**Published:** 2018-06-26

**Authors:** Alex Plata, Albina Lebedeva, Pavel Denisov, Olga Nosova, Tatiana Y. Postnikova, Alexey Pimashkin, Alexey Brazhe, Aleksey V. Zaitsev, Dmitri A. Rusakov, Alexey Semyanov

**Affiliations:** ^1^UNN Institute of Neuroscience, N. I. Lobachevsky State University of Nizhny Novgorod, University of Nizhny Novgorod, Nizhny Novgorod, Russia; ^2^Laboratory of Molecular Mechanisms of Neural Interactions, Sechenov Institute of Evolutionary Physiology and Biochemistry, Russian Academy of Sciences, St. Petersburg, Russia; ^3^Department of Medical Physics, Peter the Great St. Petersburg Polytechnic University, St. Petersburg, Russia; ^4^Department of Biophysics, Faculty of Biology, M. V. Lomonosov Moscow State University, Moscow, Russia; ^5^Institute of Experimental Medicine, Almazov National Medical Research Centre, St. Petersburg, Russia; ^6^UCL Institute of Neurology, University College London, London, United Kingdom; ^7^Department of Molecular Neurobiology, Shemyakin-Ovchinnikov Institute of Bioorganic Chemistry, Russian Academy of Sciences, Moscow, Russia; ^8^All-Russian Research Institute of Medicinal and Aromatic Plants, Moscow, Russia

**Keywords:** astrocyte remodeling, epilepsy, *D*-serine, calcium, plasticity, spatial entropy, spatial complexity, neurodegeneration

## Abstract

Epilepsy is a group of neurological disorders commonly associated with the neuronal malfunction leading to generation of seizures. Recent reports point to a possible contribution of astrocytes into this pathology. We used the lithium-pilocarpine model of *status epilepticus* (SE) in rats to monitor changes in astrocytes. Experiments were performed in acute hippocampal slices 2–4 weeks after SE induction. Nissl staining revealed significant neurodegeneration in the pyramidal cell layers of hippocampal CA1, CA3 areas, and the hilus, but not in the granular cell layer of the dentate gyrus. A significant increase in the density of astrocytes stained with an astrocyte-specific marker, sulforhodamine 101, was observed in CA1 *stratum (str.) radiatum*. Astrocytes in this area were also whole-cell loaded with a morphological tracer, Alexa Fluor 594, for two-photon excitation imaging. Sholl analyses showed no changes in the size of the astrocytic domain or in the number of primary astrocytic branches, but a significant reduction in the number of distal branches that are resolved with diffraction-limited light microscopy (and are thought to contain Ca^2+^ stores, such as mitochondria and endoplasmic reticulum). The atrophy of astrocytic branches correlated with the reduced size, but not overall frequency of Ca^2+^ events. The volume tissue fraction of nanoscopic (beyond the diffraction limit) astrocytic leaflets showed no difference between control and SE animals. The results of spatial entropy-complexity spectrum analysis were also consistent with changes in ratio of astrocytic branches vs. leaflets. In addition, we observed uncoupling of astrocytes through the gap-junctions, which was suggested as a mechanism for reduced K^+^ buffering. However, no significant difference in time-course of synaptically induced K^+^ currents in patch-clamped astrocytes argued against possible alterations in K^+^ clearance by astrocytes. The magnitude of long-term-potentiation (LTP) was reduced after SE. Exogenous *D*-serine, a co-agonist of NMDA receptors, has rescued the initial phase of LTP. This suggests that the reduced Ca^2+^-dependent release of *D*-serine by astrocytes impairs initiation of synaptic plasticity. However, it does not explain the failure of LTP maintenance which may be responsible for cognitive decline associated with epilepsy.

## Introduction

Epilepsy is a group of neurological disorders, associated with pathological synchronization of neuronal activity causing seizures. Several cellular mechanisms of this pathology were proposed. The classical view holds that seizures occur because of a shift in the balance between excitation and inhibition in the brain toward excitation (During and Spencer, 1993; DiNuzzo et al., 2014). However, pathological synchronization of excitatory neurons may also result from enhanced inhibition of a relatively small group of inhibitory interneurons, which then synchronously disinhibits numerous excitatory neurons. This is called “rebound” excitation and may make all these cells to fire in synchrony (Paz and Huguenard, 2015). The K^+^ hypothesis of epilepsy suggests that extracellular accumulation of this ion in the brain depolarizes neurons and cause epileptiform activity (Green, 1964; Fertziger and Ranck, 1970; Fröhlich et al., 2008). Mutation in astrocytic K^+^ channels (K_ir_4.1 type) has been proposed as one of the causes of human epilepsy (Villa and Combi, 2016). Indeed, it has long been reported that periodic elevations of extracellular K^+^ concentration could produce long-term changes in neuronal excitability, making the network prone to epileptogenesis (Semyanov and Godukhin, 1997).

An excessive activity of neuronal networks induces excitotoxicity, which leads to neurodegeneration. Hippocampal sclerosis is typically reported in temporal lobe epilepsy (TLE) (Kim, 2001; Blümcke et al., 2002; de Lanerolle and Lee, 2005; Thom, 2014). The loss of neurons is considered as a mechanism for epileptic focus formation and an indication for hippocampus removal in clinic. Neurodegeneration in TLE may also lead to a mild cognitive impairment affecting learning and memory (Hermann and Seidenberg, 2007; Höller and Trinka, 2014). Long-term potentiation (LTP) and depression (LTD) are widely accepted experimental models to explore mechanisms of synaptic memory formation (Bliss and Collingridge, 1993; Ju et al., 2004). Recent studies have demonstrated a significant impairment of long-term synaptic plasticity after SE in different animal models, including a lithium-pilocarpine model of TLE (Zhou et al., 2007; Müller et al., 2013; Cunha et al., 2015; Kryukov et al., 2016; Carpenter-Hyland et al., 2017; Ivanov and Zaitsev, 2017; Postnikova et al., 2017). A reduction or elimination of LTP that can be observed for weeks after SE has frequently been reported (Zhang et al., 2010; Zhou et al., 2011; Suárez et al., 2012). Despite numerous studies, the exact mechanisms of LTP impairment after SE remain unidentified.

The quest for the mechanisms of epileptogenesis have typically been focused on neuronal malfunction, such as altered expression of receptors and channels; extracellular K^+^ accumulation synchronizing neuronal populations; a shift in the balance of synaptic excitation and inhibition toward excitation (DiNuzzo et al., 2014). Intriguingly, many of these functions are regulated by astrocytes. Astrocytic uptake of neurotransmitters and extracellular K^+^ clearance are key to synaptic function (Cheung et al., 2015; Lebedeva et al., 2018; Verkhratsky and Nedergaard, 2018). Astrocytes control synaptic plasticity in glutamatergic synapses by releasing of *D*-serine, a co-agonist of NMDA receptors (Henneberger et al., 2010, 2012; Papouin et al., 2017). They are involved in uptake and release of GABA, inhibitory neuro- and gliotransmitter (Angulo et al., 2008; Lee et al., 2010; Héja et al., 2012; Kersante et al., 2013; Song et al., 2013). Therefore, astrocytic mechanisms can potentially contribute to epileptogenesis. Indeed, astrocyte control of synaptic NMDA receptors is implicated in the progressive development of TLE (Clasadonte et al., 2013). Astrocytic Ca^2+^-dependent glutamate release is suggested to trigger synchronous neuronal discharges in rat hippocampal slices following application of a K^+^ channel blocker, 4-aminopyridine (4-AP) (Tian et al., 2005). In contrast, some other models of acute epileptiform activity in slices (Mg^2+^-free solution, picrotoxin, increased extracellular K^+^) produce Ca^2+^ oscillations in astrocytes, which are not responsible for paroxysmal activity in neurons (Fellin et al., 2006). Thus, the role of astrocytic Ca^2+^ activity in epileptogenesis remains controversial. Moreover, acute effects have often been obtained in brain slices using convulsants, which may not necessarily reflect changes in astrocytic Ca^2+^ activity after SE.

Another critical aspect of neuron-astrocyte interactions is morphological. Astrocytic processes approach synapses, forming so-called “astrocytic cradle” (Verkhratsky and Nedergaard, 2014). Being highly plastic, perisynaptic astrocytic processes can retract from or extend toward dendritic spines (Bernardinelli et al., 2014a; Heller and Rusakov, 2015). Rearing laboratory animals in complex environment or certain LTP induction protocols appear to increase glial coverage of excitatory synapses (Jones and Greenough, 1996; Lushnikova et al., 2009) whereas some memory consolidation tasks (Ostroff et al., 2014) or experiencing a lactation period (Oliet et al., 2001) appear to decrease it. Suppression of IP_3_-dependent Ca^2+^ signaling in astrocytes reduces synaptic coverage (Tanaka et al., 2013). Morphological remodeling of astrocytes following SE has not been systematically studied.

Impairment of K^+^ clearance by astrocytes has been proposed as a mechanism for extracellular K^+^ accumulation in the epileptic brain (Bedner and Steinhauser, 2013). This may occur due to the redistribution of astrocytic K^+^ channels or reduced K^+^ buffering due to astrocyte uncoupling through gap-junctions in epileptic tissue (Wallraff et al., 2006; Bedner et al., 2015). Interestingly, K^+^ clearance by astrocytes can be linked to astrocytic Ca^2+^ activity, via Ca^2+^-dependent K^+^ channels (Wang et al., 2012). However, this link has not been considered in the context of epileptogenesis.

Although recent reports have convincingly demonstrated the involvement of astrocytes in epileptogenesis, further analysis of cellular and subcellular mechanisms is still needed. Here we report morphological and physiological changes in astrocytes following SE induced by lithium-pilocarpine injection, as well as their possible association with synaptic plasticity changes. Our quantitative morphological assessment employs a novel approach based on the spatial entropy-complexity spectrum analysis.

## Materials and methods

### Pilocarpine model of epilepsy

All procedures were carried out in accordance with University of Nizhny Novgorod regulations. 3–6 weeks old male Sprague-Dawley (Wistar for LTP experiments) rats were injected with lithium chloride (127 mg/kg, Sigma Aldrich) 20–24 h prior to pilocarpine and methylscopolamine (1 mg/kg, Sigma Aldrich) 20 min prior to pilocarpine. Then pilocarpine (Tocris), 10 mg/kg was injected every 30 min (but no more than 60 mg/kg) to induce SE which characterized with generalized seizures lasting for at least 20 min (Supplementary Figure 1). To reduce mortality, phenazepam 1 mg/kg was injected every 10 min for 30–40 min after 20 min of generalized seizures.

### Nissl staining

Brain tissue was prepared according to routine histologic methods (Singh et al., 2008). Briefly, brains were removed immediately after decapitation, immersed in ethanol 96% and embedded in paraffin after dehydration. Paraffin sections (5 μm) were cut in a coronal plane and stained with Nissl's method. Each sixth staining section was chosen for quantitative analysis for each animal. Images of CA1, CA3, hilus, and dentate gyrus were obtained using an x40 magnification. The neurons were counted per 100 μm for cell layer in each area using the plugin “Cell counter” for ImageJ.

### Hippocampal slice preparation

The slices were prepared 2–4 weeks after SE. The animals were anesthetized with Isoflurane (1-Chloro-2,2,2-trifluoroethyl difluoromethyl ether) and then decapitated. The rest of the procedure was slightly different for whole cell and field potential recordings.

#### Preparation for whole-cell recording and imaging

The brains were exposed, and then chilled with ice-cold solution containing (in mM): 50 sucrose; 87 NaCl; 2.5 KCl; 8.48 MgSO4; 1.24 NaH_2_PO_4_; 26.2 NaHCO_3_; 0.5 CaCl_2_; 22 D-Glucose. Hippocampi from both hemispheres were dissected, isolated, and transverse slices (350 μm) were cut using a vibrating microtome (Microm HM650 V; Thermo Fisher Scientific) and left to recover at 34°C for 1 h in a submerged incubation chamber with “storage” solution containing (in mM): 119 NaCl; 2.5 KCl; 1.3 MgSO_4_; 1 NaH_2_PO_4_; 26.2 NaHCO_3_; 1 CaCl_2_; 1.6 MgCl_2_; 22 D-Glucose. Then the slices were transferred to the recording chamber and were continuously perfused with a solution containing (in mM): 119 NaCl; 2.5 KCl; 1.3 MgSO_4_; 1 NaH_2_PO_4_; 26.2 NaHCO_3_; 2.5 CaCl_2_; 11 D-Glucose. All solutions were saturated with carbogen gas mixture containing 95% O_2_ and 5% CO_2_. Osmolarity was 295 ± 5 mOsm, pH 7.4. All recordings were done at a temperature of 34°C.

#### Preparation for field potential recording and LTP induction

The cerebellum and a small section of the frontal cortex were removed. A flat surface for mounting the brain was created by making a cut on the dorsal surface parallel to the horizontal plane. The brain was then mounted onto the stage of the vibratome, and horizontal sections (400 μm thick) were cut in ice-cold artificial cerebrospinal fluid (ACSF). ACSF composed of (in mM): 126 NaCl, 2.5 KCl, 1.25 NaH_2_PO_4_, 1 MgSO_4_, 2 CaCl_2_, 24 NaHCO_3_, and 10 D-glucose was saturated with carbogen. The prepared slices were immersed in a chamber with ACSF, which was placed in a temperature-controlled water bath (35°C) for 1 h. After the incubation, the slices were transferred to the recording chamber, where they were kept for 15–20 min prior to the electrophysiological study. In this chamber, hippocampal slices were perfused with a constant flow of oxygenated ACSF at a rate of 5 ml/min at room temperature. One to five slices from each rat were used in the experiment.

### Sholl analysis

Sholl analysis was performed on adaptively thresholded maximal projections of Z-stacks, where each XY-plane has been filtered with anisotropic diffusion filtering. All processing steps were performed using image-funcut library [image-funcut, https://github.com/abrazhe/image-funcut] and other custom-written Python scripts, using Scikit-Image [scikit, http://scikit-image.org/] and Sci-Py [scipy, http://www.scipy.org/] libraries (Van Der Walt et al., 2014). The step-by-step procedure is summarized in Supplementary Figure 2. Sholl metric was calculated automatically as a number of intersections of circles with centers at the soma and increasing radii with the thresholded mask obtained as described above.

### Shearlet-based estimate of spatial complexity and entropy for 2D patterns

A spatial pattern can be characterized by a pair of statistical properties, namely entropy and statistical complexity (López-Ruiz et al., 1995). An ordered (e.g., periodic) structure with a single spatial scale and preferred feature orientation will have both low entropy and small statistical complexity, as the structure in any part of the system can be reconstructed from a small area. At the other end of the complexity-entropy spectrum, where the state is disordered with no spatial correlations, the entropy of the system will be maximal, while the complexity will again be low (the spatial pattern has the same local statistics). Intermediate cases with high statistical complexity are of more interest, as they represent systems with non-trivial regularities and underlying structure embedded in randomness. We developed an algorithm to map local entropy and complexity values for biologically relevant structures using shearlet transform to induce local probability densities of scale and orientation and Jensen-Shannon divergence to define statistical complexity. Below we describe the two points in more detail.

#### Entropy and statistical complexity

Both entropy and complexity (entropic non-triviality) measures for a 2D pattern were defined statistically for a distribution of spatial features, such as orientation, or scale. Here we denoted such a distribution as

(1) $$\begin{array}{l} P:=\left\{P_{i}\right\} \end{array}$$

for a set of features *i* = 1 … *N*. Then entropy was defined simply as Shannon information entropy

(2) $$\begin{array}{l} S\left[P\right]=-\underset{i}{\sum }P_{i}\;log_{2}\;P_{i}. \end{array}$$

Entropy will have its maximum for the equiprobable distribution of all features *P*_*e*_,

(3) $$\begin{array}{l} S\left[P_{e}\right]=S_{\max}=2N, \end{array}$$

where *N* is a number of possible states or features. This allows to introduce normalized entropy:

(4) $$\begin{array}{l} H_{s}\left[P\right]:=S\left[P\right]/S\left[P_{e}\right],H_{s}\left[P\right]\in \left[0,\ldots 1\right] \end{array}$$

Following (López-Ruiz et al., 1995) we used the disequilibrium-based complexity measure

(5) $$\begin{array}{l} C\left[P\right]:=Q\left[P,P_{e}\right]H_{s}\left[P\right] \end{array}$$

i.e., the one based on the statistical distance between the observed (*P*) and equiprobable (*P*_*e*_) distributions. Here, following (Lamberti et al., 2004; Rosso et al., 2007), we employed normalized Jensen-Shannon divergence

(6) $$\begin{array}{l} Q_{JS}=J\left[P,P_{e}\right]/J_{\max} \end{array}$$

as a measure of distance between two distributions, where Jensen-Shannon divergence is defined as

(7) $$\begin{array}{l} J\left[P,P_{e}\right]=S\left[\frac{P+P_{e}}{2}\right]-\frac{1}{2}\left(S\left[P\right]+S\left[P_{e}\right]\right). \end{array}$$

Clearly, *J*[*P, P*_*e*_] = 0 if *P* = *P*_*e*_ and reaches its maximum when only one feature, say *m*, is present, while all others are absent: *P*_*i*_ = 1|*i* = *m*, and *Pi* = 0|*i* ≠ *m*.

#### Shearlet transform

Shearlet transform provides a convenient probability density function for spatial entropy and complexity estimates, describing local prevalence of structures with some specific scale and orientation. We used fast finite discrete shearlet transform (FFST) described in detail by (Häuser and Steidl, 2013). Here we provide a minimally sufficient description of the FFST and its use in calculation of spatial entropy and complexity.

Discrete shearlet transform was based on convolving the digital 2D image

(8) $$\begin{array}{l} I\left(x,y\right)\in R^{\left(N,N\right)} \end{array}$$

with scaled, sheared, and shifted copies of a “mother” shearlet function ψ, thus accounting for different scales and orientations of features contained in the image; one uses the dilation matrix A and shear matrix S to create the sheared, scaled and shifted copies of the mother wavelet ψ_*x*_:

(9) $$\begin{array}{l} \psi_{a,s,t}=a^{-3/4}\left(Aa^{-1}Ss^{-1}\left(x-t\right)\right). \end{array}$$

Thus, the scaled and shared copies of ψ pick up dominant anisotropic features at different spatial scales and orientations. In the discrete transform, one uses a fixed number of decomposition scales and shifts as well as scale-dependent number of orientations (more orientations at higher spatial frequencies). Finally, shearlet decomposition of image was given by shearlet coefficients

(10) $$\begin{array}{l} T\left(I\right)\left(j,k,m\right)=\langle I,\psi_{j,k,m}\rangle \end{array}$$

where discrete shearlet ψ_*j,k,m*_ = ψ_*ajsj,ktm*_(*x*) is the shearlet at discrete scale α_*j*_, shear *s*_*j,k*_ and shift *t*_*m*_. Thus, *T(I)* is a set of *K* images of the same size as *I(x,y)*, where the value at a specific *(x,y)* location in the *k*-th image represents the shearlet coefficient at some specific scale *j* and shear *s*.

Following ideas from wavelet entropy (Rosso et al., 2001) and earlier of spectral entropy of (Powell and Percival, 1979), in each location of the studied 2D image *I(x,y)*, we defined *P*(*x, y*) = *Pk*(*x, y*) as normalized power of the shearlet coefficients at this point:

(11) $$\begin{array}{l} P_{k}\left(x,y\right)=E_{k}\left(x,y\right)/\sum_{j}E_{j}\left(x,y\right) \end{array}$$

thus, interpreting a spectrum of local feature scales and orientations as a density function. Here $\left(K_{\sigma }^{*}\cdot \right)$ denotes convolution with a Gaussian kernel with scale-dependent standard deviation σ_*j*_.

### Volume fraction (VF) of astrocytic leaflets

To calculate the VF of the fine process of the astrocyte, we followed a similar method described by Heller and Rusakov (2015) A line of 45 μm length were drawn from the soma on a single Z plane of the stack. Spatial attention was paid to ensure that fluorescence of soma was not saturated. The estimated VF was calculated with the following:

(12) $$\begin{array}{l} GV\left(i,j\right)=\left(F\left(i,j\right)-F_{0}\right)/\left(F_{max}-F_{0}\right) \end{array}$$

where *F(I,j)*—the fluorescent in particular pixel of the line, *F*_*max*_—the fluorescence of soma, F_0_–the background fluorescence. F_0_ was obtained in image area which had no stained astrocytes.

### Astrocyte coupling analysis

The astrocytes were loaded with 50 μM Alexa Fluor 594 through the patch pipette for 30 min. then Z-stack two-photon images was obtained (emission band-pass filter 565–610 nm, 512 x 512 pixels). The images were then denoised with block matching 4D (BM4D) free scrip for MATLAB (Maggioni et al., 2013; Danielyan et al., 2014). The distance to neighboring astrocytes coupled to the target astrocyte through gap-junctions was calculated in 3D-space using Pythagorean theorem with custom-written MATLAB script. Fluorescence Intensities of all coupled cells were normalized to fluorescence of soma of the patched astrocyte. The relationship between distance fluorescence of coupled astrocyte and distance to this astrocyte was fitted by monoexponential function to obtain coupling length constant (*C*_λ_*)* (Anders et al., 2014):

(13) $$\begin{array}{l} I\left(d\right)=I_{0}exp\left(-d/C_{\lambda }\right), \end{array}$$

were, d—distance, I_o_—the normalized fluorescence intensity of the coupled cell.

### Electrophysiological recordings

#### Whole-cell recording

Whole-cell voltage-clamp and current-clamp were performed with Multiclamp 700B amplifier (Molecular Devices). The CA1 *str.radiatum* astrocytes were visualized with BX51WI (Olympus) or Axio Examiner Z1 (Zeiss) microscope equipped with infrared differential interference contrast. Borosilicate patch pipettes (Resistance 3−5 MΩ) were filled with internal solution containing (in mM): 130 KCH_3_SO_3_, 10 HEPES, 10 Na_2_-phosphocreatine, 8 NaCl, 3 l-ascorbic acid, 2 Mg-GTP (pH adjusted to 7.2, osmolarity of 295 ± 3 mOsm). For simultaneous two-photon imaging, 50 μM Alexa Fluor 594 was added to the internal solution.

Bipolar extracellular tungsten electrode (FHC) was placed in *str. radiatum* between CA1 and CA3 areas. Once whole-cell configuration was obtained, the cell was dialyzed for 5 to 10 min before the start of recording. In voltage clamp recordings the astrocytes were held at−80 mV. Voltage steps were applied to obtain current-voltage (I-V) relationship. In current clamp, current steps were applied to corroborate the absence of membrane excitability. Cycles of 1, 4, and 5 electrical stimuli (100 ms, 50 Hz) were applied to Schaffer collaterals. The intensity of stimulation was set to induce synaptic currents in astrocytes of 20 to 50 pA to a single stimulus. Series and input resistances were continuously monitored by a voltage step of−5 mV after each cycle. Signals were sampled at 5 kHz and filtered at 2.5 kHz.

Passive astrocytes were taken at 100−200 μm from the stimulating electrode. They were identified by small soma (5–10 μm), low resting membrane potential (~−80 mV), low input resistance (<20 MΩ), and linear I-V relationship. Cells with similar characteristics except for higher input resistance (>50 MΩ) were considered NG2 or complex cells and were excluded from the study.

Membrane currents were analyzed using custom-written MATLAB scrips (MathWorks R2016a). Synaptic currents of 1, 4, and 5 stimuli were baseline subtracted and then averaged. I_K_ (K^+^ current) was measured 200 ms after the stimulus. At this time point I_K_ was not contaminated by the current mediated by field potential and transporter current. From this point the decay was fitted with mono-exponential function and τ_decay_ calculated. To obtain I_K_ in response to 5th stimulus, the response to 4 stimuli was subtracted from the response to 4 stimuli.

#### Field potential recording

Field excitatory postsynaptic potentials (fEPSPs) were recorded from CA1 *str. radiatum* using glass microelectrodes (0.2–1.0 MΩ) filled with ACSF. Synaptic responses were evoked with extracellular stimulation of the Schaffer collaterals using a bipolar twisted stimulating electrode made of insulated nichrome wire placed in the *str. radiatum* at the CA1–CA2 border. The stimulation was performed with rectangular paired pulses (duration, 0.1 ms; interstimulus interval, 50 ms) every 20 s via an A365 stimulus isolator (WPI). Responses were amplified using a microelectrode AC amplifier model 1800 (A-M Systems) and were digitized and recorded on a personal computer using ADC/DAC NI USB-6211 (National Instruments) and WinWCP v5.2.2 software by John Dempster (University of Strathclyde). Electrophysiological data were analyzed with the Clampfit 10.2 program (Axon Instruments).

The dependence of field response amplitude on stimulation strength was determined by increasing the current intensity from 25 to 300 μA. For each fEPSP, the amplitude and the slope of the rising phase at a level of 20–80% of the peak amplitude were measured. The presynaptic fiber volley (PrV) was quantified by the peak amplitude. The maximum rise slope of the input-output (I/O) relationships (fEPSP amplitude vs. PrV amplitude) was calculated for every slice by fitting with a sigmoidal Gompertz function (Equation 14) using OriginPro 8 (OriginLab Corporation).

(14) $$\begin{array}{l} y=ae^{{-e}^{\left(-k\left(x-x_{c}\right)\right)}}, \end{array}$$

where *a* is an asymptote of the maximum fEPSP amplitude; *e* is Euler's Number (*e* = 2.71828 …); *k* and *x*_*c*_ are positive numbers describing the shape of the curve; *x*_*c*_ is the PrV amplitude at which the maximum slope of the curve is observed; *ak*/*e* is a maximum slope of the curve.

The stimulus intensity used in the experiment was chosen so that the amplitude of fEPSP would be 40–50% of the amplitude where the population spike appeared for the first time. The strength of stimulation was unvaried during the experiments, usually being 50–150 μA. The paired-pulse ratio (PPR) was measured as a ratio of the second and first fEPSP amplitude.

The LTP induction was started only if a stable amplitude of the baseline fEPSP had been recorded for 20 min. Three trains of high-frequency stimulation (HFS, 100 pulses at 100 Hz, with an inter-train interval of 20 s protocol) was applied to induce LTP. The fEPSPs were recorded after induction protocol during 60 min. The baseline fEPSP and the potentiated fEPSP (recorded 47–60 min after HFS) were averaged separately to measure LTP in a slice. Plasticity value was calculated as a ratio of the slope of the rising phase in the averaged potentiated and baseline fEPSP.

### Ca^2+^ imaging

Ca^2+^ activity was recorded with a confocal microscope, Zeiss LSM DuoScan 510, in CA1 *str.radiatum* of acute hippocampal slices pre-incubated with Ca^2+^ dye, Oregon Green 488 BAPTA-1 AM (Invitrogen) and an astrocyte specific marker, sulforhodamine 101 (100 nM, Invitrogen). After the preparation, the slices were transferred to a 3 ml incubation chamber with constantly gassed ACSF containing both dyes. Oregon Green 488 BAPTA-1 AM was initially dissolved to 0.795 mM in 0.8% Pluronic F-127 in DMSO. Then 3 μl of the dye was added to the chamber. After incubation for 40–45 min at 37°C in the dark, the slices were transferred to the recording/imaging chamber for time-lapse imaging (one frame/s). Oregon Green 488 BAPTA1 was excited with a 488 nm argon laser and imaged with an emission band-pass filter 500–530 nm; sulforhodamine 101 was excited with a 543 nm HeNe laser and imaged with an emission band-pass filter 650–710 nm. The imaging was performed for 10 min at 34°C in normal ASCF, then 30 dark noise images were recorded.

The raw imaging data were exported to MATLAB. The median of the dark noise was calculated for each pixel and subtracted from the corresponding pixel intensity value of the fluorescence images. Then denoising was done with the BM3D algorithm (Danielyan et al., 2014). The movement artifacts were corrected with the single-step DFT algorithm (Guizar-Sicairos et al., 2008). The whole Ca^2+^ events (x-y-time 3D Ca^2+^ signals) were detected with the adapted algorithm which we described previously (Wu et al., 2014). Briefly, each pixel of the image series was analyzed independently. Firstly, we roughly estimated a baseline fluorescence F_0temp_ applying 60-s 3rd order Savitzky-Golay polynomial filter which smoothed all Ca^2+^ signals on the fluorescent signal F. Then, we estimated a temporary (ΔF/F)_temp_ = (F − F_0temp_) / F_0temp_ to find Ca^2+^ transients exceeding a statistical threshold. Then these transients were excluded from the baseline which was further smoothed with 100-s filter. This filter interpolated the intervals left by the excluded transients, and, thus, we obtained the uninterrupted final baseline F_0_ which was used to obtain ΔF/F = (F − F_0_) / F_0_. Ca^2+^ transients exceeding a statistical threshold were detected and binarized. The active neighboring pixels were grouped into x-y 2D Ca^2+^ events, which were reconstructed into x-y-time 3D Ca^2+^ events. For each Ca^2+^ event the maximal projection (S_max_), the integral and the duration were calculated. To avoid noise detection the events excluded from further analysis if the integral was less than 4 μm^2^ s, or the S_max_ was less than 10 μm^2^, or the duration less than 2 s.

The probability density of the events sizes and the durations appeared linear in log-log scale, suggesting that the obtained distributions can be described by a power law. Therefore, the power law fit was applied, and the corresponding exponent was calculated for each slice.

### Statistical analysis

All data are presented as mean ± standard error of mean (SEM). Statistical significance was assessed using non-parametric Mann-Whitney test, parametric Student's *t*-test and repeated measures two-way ANOVA as stated in the text. *P* < 0.05 was considered statistically significant.

## Results

**Figure 1:**
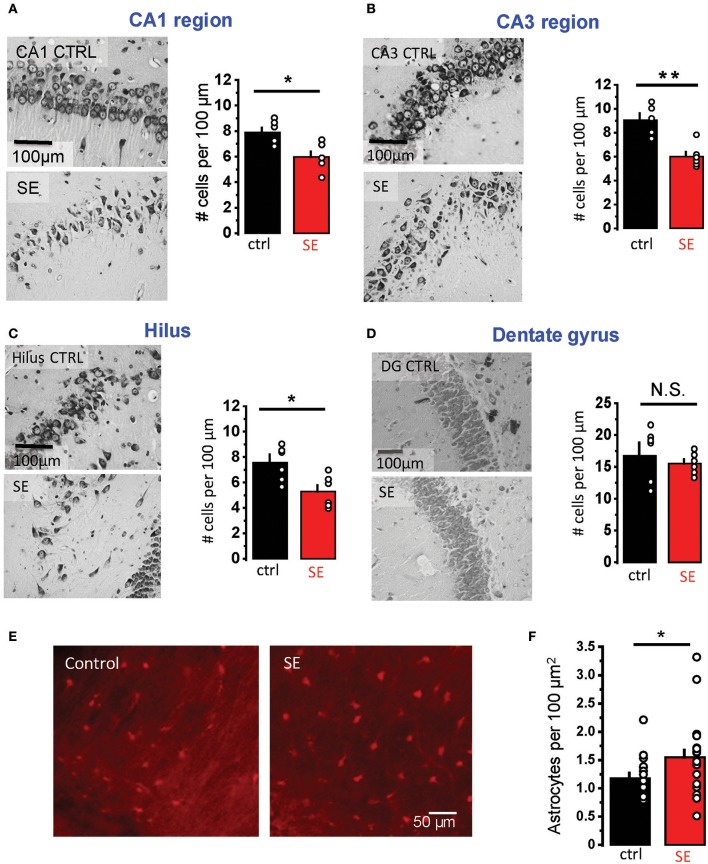
Neurodegeneration and astrogliosis after SE. **(A–D)** Nissl staining showing neurodegeneration in the *str. pyramidale* of CA1 **(A)**, CA3 **(B)**, and hilus **(C)** after SE. No significant neurodegeneration was observed in the granular cell layer of dentate gyrus **(D)**. *Right*, illustrations of stained cells in control (*top*) and SE-rats (*bottom*). *Left*, the summary data from several rats. The cells were counted along the cell layers and normalized to the length of the layers. **(E)** Fluorescent images of astrocytes stained with sulforhodamine 101: in control (*left image*) and SE-rats (*right image*). **(F)** the summary data on several rats. The circles show values in individual rats. The bars with error bars are means ± SEMs. **p < 0.01, *p < 0.05, N.S. *p* > 0.05 Mann-Whitney test.

Rat hippocampal slices were prepared 2–4 weeks after pilocarpine-induced SE when the animals typically started to develop spontaneous seizures. Nissl staining confirmed statistically significant neurodegeneration in pyramidal layers of three hippocampal regions: CA1, CA3, and hilus (CA1: 7.9 ± 0.36 cells per 100 μm in control, *n* = 6; 6.0 ± 0.4 cells per 100 μm after SE, *n* = 6; *p* = 0.02; CA3: 8.9 ± 0.5 cells per 100 μm in control, *n* = 6; 6.0 ± 0.4 cells per 100 μm after SE, *n* = 6; *p* = 0.01, Mann-Whitney test; hilus: 7.5 ± 0.6 cells per 100 μm in control, *n* = 6; 5.3 ± 0.5 cells per 100 μm after SE, *n* = 6; *p* = 0.04, Mann-Whitney test; Figures 1A–C). No significant changes in the cell density were observed in granular cell layer of the dentate gyrus (17.2 ± 1.7 cells per 100 μm in control, *n* = 6; 15.5 ± 0.7 cells per 100 μm after SE, *n* = 6; *p* = 0.37, Mann-Whitney test; Figure 1D). Consistent with previously reported astrogliosis, the density of CA1 *str. radiatum* astrocytes stained with an astrocyte-specific marker, sulforhodamine 101, was significantly higher after SE (1.2 ± 0.1 cells per 100 μm^2^ in control, *n* = 15; 1.5 ± 0.1 cells per 100 μm^2^ after SE, *n* = 20; *p* = 0.02, Mann-Whitney test; Figures 1E,F) (Mazzuferi et al., 2010; Pekny et al., 2016). However, this astrogliosis would be considered rather minor.

**Figure 2:**
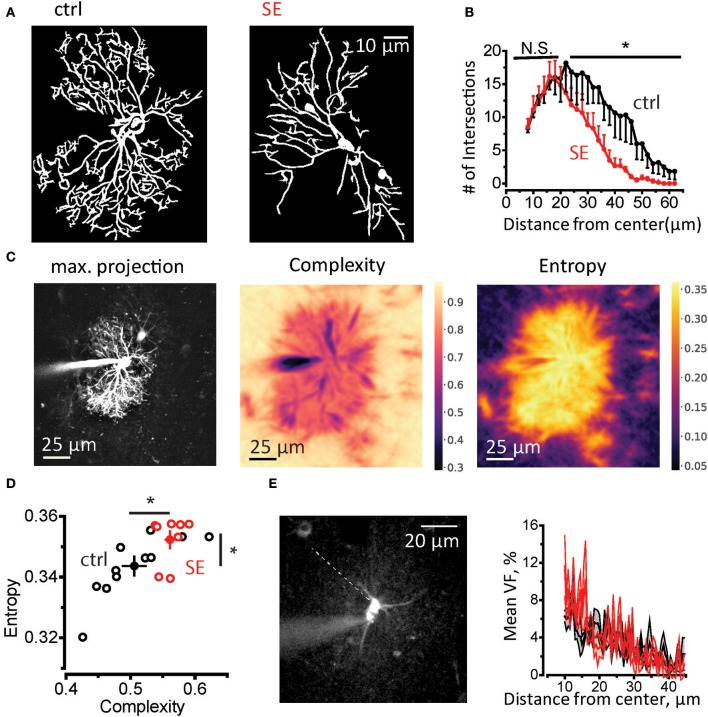
Morphological remodeling of astrocytes after SE. **(A)** Masks of astrocytic branches in control (left) and SE rats (right) which were used for Sholl analysis. The masks were obtained from maximal projections of z-stack of fluorescence images of astrocytes loaded with 50 μM Alexa Fluor 594 through patch pipette. **(B)** The summary data for the number of intersections of circles drawn around center of the astrocyte soma with astrocytic branches. *p < 0.05, N.S. *p* > 0.05, two-way ANOVA. **(C)** Spatial entropy-complexity analysis. *Left*, maximal projection of Z-stack of fluorescence images of an astrocyte. *Middle*, The spatial complexity profile of the astrocyte. *Right*, The spatial entropy profile of the astrocyte. **(D)** The summary graph of spatial entropy-complexity pairs of astrocytes on control (black circles) and SE-rats (red circles). Empty circles are individual astrocytes. Filled circles are means ± SEMs. **(E)** Estimation of astrocyte leaflets' VF. *Left*, construction of fluorescence profile across an astrocyte. Dashed line indicates the place there the profile was obtained. It passes through the soma and the area or unresolved processes devoid of identifiable branches. *Right*, the mean ± SEM fluorescent profiles normalized to the fluorescence in soma for control (*black trace*) and SE-rats (*red trace*).

Then we performed Sholl analysis on two-photon images of astrocytes loaded trough patch pipette with 50 μM Alexa Fluor 594 (morphological tracer, see Materials and Methods, Supplementary Figure 2 and Figure 2A). There was no significant difference in the number of primary branches (connected to soma), the peak number of the branches and size of the astrocytic domain in control and SE animals (Supplementary Figure 3). However, the number of distal branches was significantly lower after SE (control, *n* = 6; SE, *n* = 6; two-way repeated measures (RM) ANOVA, *F*_(1, 5)_ = 6.862, *p* = 0.047, partial η^2^ = 0.578, with a mean difference of 5.61 ± 2.14; Figure 2B). This morphological rearrangement can also be assessed using the analysis of spatial complexity-entropy spectrum (Figure 2C). Spatial complexity and spatial entropy are both low in highly ordered or anisotropic systems. As the system loses the order, both entropy and complexity start to increase. When the elements of the systems become randomly distributed (“noise”) the entropy is the highest, while the complexity decreases. Remodeling of astrocytic processes after SE significantly increased both entropy and complexity (entropy: 0.51 ± 0.02 in control, *n* = 11; 0.56 ± 0.001 after SE *n* = 8; *p* = 0.009, Mann-Whitney test; complexity 0.343 ± 0.003 in control; 0.352 ± 0.003 after SE; *p* = 0.02, Mann-Whitney test; Figure 2D). This finding suggests that astrocytic processes become less orderly organized after SE. It can be potentially explained by the decrease in the ratio of primary astrocytic branches which are resolved with diffraction-limited light microscopy and thin astrocytic leaflets which appear as a chaotic fluorescent pattern because of their sizes are beyond diffraction-limited light microscopy resolution. To estimate possible changes in leaflets volume fraction, we performed line scan through soma and the area of unresolved astrocytic leaflets avoiding astrocytic branches (Figure 2E). This method is based on the assumption that unsaturated fluorescence level in soma corresponds to 100% volume fraction (VF) (Medvedev et al., 2014; Heller and Rusakov, 2015). The ratio between the fluorescence of leaflets area and fluorescence of soma was considered VF of leaflets and did not differ between control and SE rats (mean leaflets VF: 3.4 ± 0.2%, *n* = 6 in control; 3.6 ± 0.2%, *n* = 6 after SE; *p* = 0.59, Mann-Whitney test; Figure 2E). The equal VF of leaflets does not, however, rule out their spatial rearrangement after SE which can be only assessed with super-resolution light microscopy or electron microscopy.

**Figure 3:**
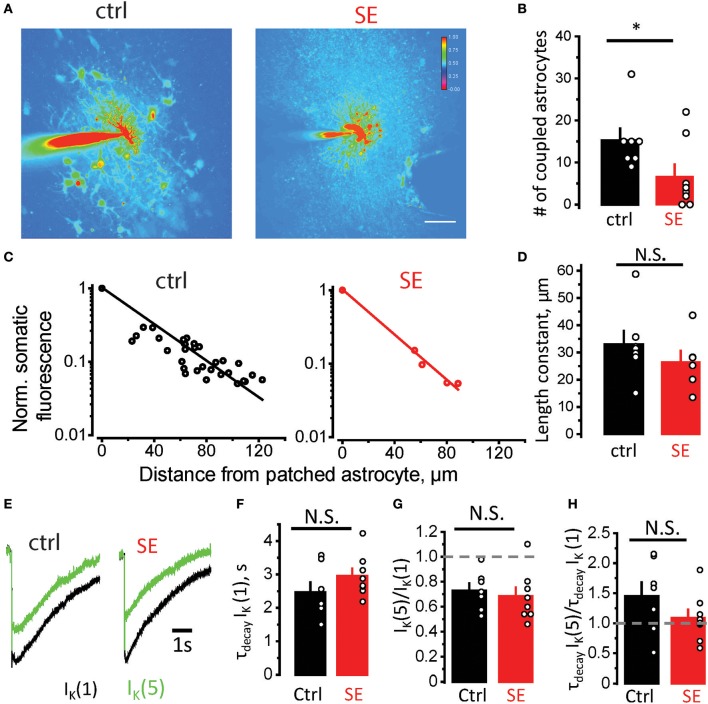
Astrocytic uncoupling through the gap-junctions does not affect K^+^ clearance by astrocytes. **(A)** Fluorescence image of an astrocyte stained with 50 μM Alexa Fluor 594 through patch pipette in control (*left*) and SE-rats (*left*). Because the dye goes through gap-junctions, the coupled astrocytes are also stained. The number of stained astrocytes is lower after SE. **(B)** The summary data showing the number of coupled astrocytes in control (*black*) and after SE (*red*). **(C)** Decay of fluorescence in somas of coupled astrocytes with distance from the astrocyte loaded with Alexa Fluor 594. The slope of the linear fit in semi-logarithmic scale determines the length constant. **(D)** The summary data showing the length constant in control (*black*) and after SE (*red*). **(E)** The sample traces of the I_K_ recorded to the single stimulus [I_K_(1), black trace] and to the fifth stimulus [the response to 4 stimuli was subtracted from the response to 5 stimuli, I_K_(5), green trace]. **(F)** The summary data of decay time constant of I_K_(1) [τ_decay_ I_K_(1)] in control (*black*) and after SE (*red*). **(G,H)** The summary data of I_K_(5)/I_K_(1) and τ_decay_ I_K_(5)/ τ_decay_ I_K_(1) ratios, respectively. The circles show values in individual rats. The bars with error bars are means ± SEMs. *p < 0.05, N.S. *p* > 0.05 Mann-Whitney test.

Morphological remodeling of astrocytes can be linked to astrocytic uncoupling and disruption of astrocytic syncytium after SE (Wallraff et al., 2006; Bedner et al., 2015). To assess astrocyte coupling, we monitored diffusion of Alexa Fluor 594 from patched astrocyte to its neighbors through gap-junctions (Figure 3A). The number of stained astrocytes was significantly lower after SE compared to control (15.3 ± 2.8, *n* = 7, in control; 6.6 ± 2.9, *n* = 8 after SE; *p* = 0.03 Mann-Whitney test; Figure 3B). We also estimated the “strength” of gap-junction connections by the decay of fluorescence measured in somas of coupled astrocytes as a function of distance from the astrocyte loaded with Alexa Fluor 594 through patch pipette. The exponential decay of fluorescence was observed both in control and SE rats (linear relationship in semi-logarithmic scale, Figure 3C). Although length constant (λ) tended to be smaller after SE, the difference did not reach significance (λ: 33 ± 5 μm, *n* = 7, in control; 26 ± 5 μm, *n* = 6, after SE, *p* = 0.07, Mann-Whitney test, Figure 3D).

Decreased coupling of astrocytes through gap-junctions can lead to reduced spatial buffering of K^+^ released during synaptic transmission (Wallraff et al., 2006; Shih et al., 2013; Bedner et al., 2015; Cheung et al., 2015; Lebedeva et al., 2018). To address possible changes in K^+^ dynamics after SE, we recorded K^+^ current (I_K_) in CA1 *str.radiatum* astrocytes in response to stimulation of Schaffer collaterals (Figure 3E). The astrocytic response consists of three overlapping currents: field potential-induced current, glutamate transporter current, and I_K_ (Sibille et al., 2014). First two currents are short and typically end within 100 ms, while I_K_ lasts for several seconds (Afzalov et al., 2013; Lebedeva et al., 2018). Therefore, the amplitude of I_K_ was measured 200 ms after the stimulus. The I_K_ was also fitted from this point with mono-exponential function, which was used to calculate the decay time-constant (τ_decay_ I_K_). No significant difference was observed in τ_decay_ I_K_ in response to single stimulus after SE [τ_decay_ I_K_(1): 2.5 ± 0.3 s, *n* = 7, in control; 3.0 ± 0.2 s, *n* = 8, after SE; *p* = 0.09, Mann-Whitney test, Figure 3F]. This result suggests that K^+^ clearance is not affected during single synaptic events after SE, but it may be affected during repeated activity. To address this issue, we stimulated Schaffer collaterals 4 times and 5 times at 50 Hz. Then the response to 4 stimuli was subtracted from the response to 5 stimuli to obtain isolated I_K_ to 5th stimulus [I_K_(5)].The ratio of I_K_(5)/I_K_(1) demonstrated activity-dependent depression of I_K_ and was not significantly different between control and SE rats [I_K_(5)/I_K_(1): 0.72 ± 0.06, *n* = 7, in control; 0.68 ± 0.07, *n* = 8, after SE; *p* = 0.26, Mann-Whitney test, Figure 3G]. The ratio of τ_decay_ I_K_(5)/τ_decay_ I_K_(1) was also not significantly different between control and SE rats [τ_decay_ I_K_(5)/τ_decay_ I_K_(1): 1.45 ± 0.23, *n* = 7, in control; 1.09 ± 0.14, *n* = 8, after SE; *p* = 0.14, Mann-Whitney test, Figure 3H]. These findings suggest that possible reduction in K^+^ spatial buffering in astrocytic syncytium does not affect K^+^ clearance during moderate activity of the neuronal network.

**Figure 4:**
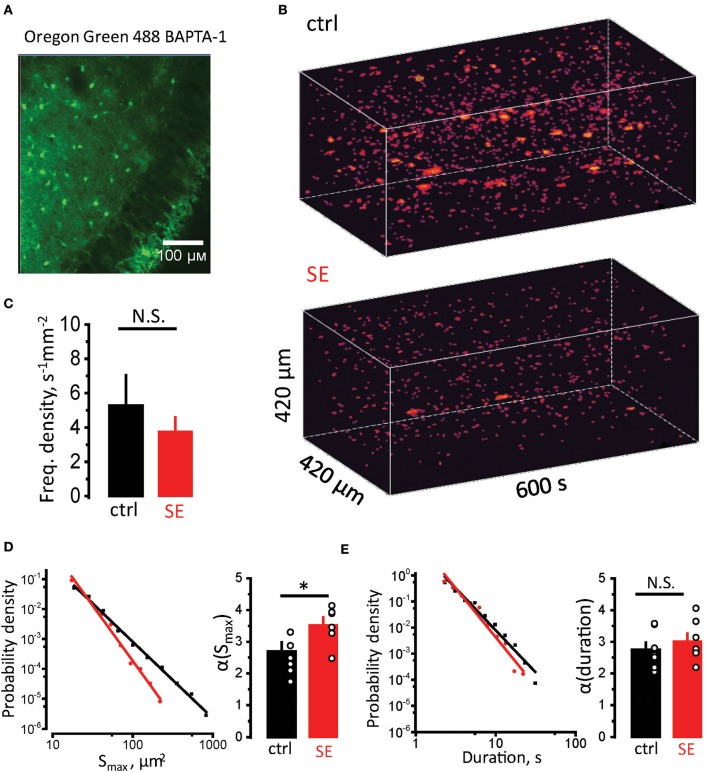
Reduction in sizes of spontaneous Ca^2+^ events in astrocytic syncytium. **(A)** Hippocampal slice stained with Oregon Green 488 BAPTA-1 AM. The image shows that astrocytes in *str.radiatum*, but not neurons in *str.pyramidale* stained. **(B)** 3D reconstruction of Ca^2+^ events in *str.radiatum* astrocytes (x-y-time). *Top*, Time-course of Ca^2+^ events in control slice. *Bottom*, in slice after SE. **(C)** The summary data of the frequency density of astrocytic Ca^2+^ events in control (black) and SE-rats (red). **(D,E)** *Right*, Probability density distributions of S_max_ **(D)** and durations **(E)** of Ca^2+^ events in log–log scale (log-binned data) for all recorded slices. Solid black and red lines are the power function fits for control and SE data, respectively. *Left*, The summary data of the exponents α(S_max_) **(D)** and α(durations) **(E)**. The circles show values in individual rats. The bars with error bars are means ± SEMs. *p < 0.05, N.S. *p* > 0.05 Mann-Whitney test.

Astrocytic atrophy and uncoupling can affect Ca^2+^ signaling in an astrocytic syncytium. To test this hypothesis, we measured Ca^2+^ signals in CA1 *str. radiatum* stained with membrane-permeable Ca^2+^ dye Oregon Green 488 BAPTA-1, AM (Figure 4A). Although this dye predominantly stains astrocytes, we could not rule out the contribution of neuronal responses to a fluorescent signal. Fortunately, astrocyte can generate much slower Ca^2+^ signals than neurons (seconds vs. hundreds of milliseconds) (Bazargani and Attwell, 2016). Such long signals are likely to represent only a proportion of overall Ca^2+^ activity in astrocytes and require Ca^2+^ release from endogenous Ca^2+^ stores. Thus, astrocytic Ca^2+^ events can be separated from neuronal based on their duration (Monai et al., 2016). Time-lapse Ca^2+^ imaging was performed with a confocal microscope at the rate of 1 frame per second. Events lasting ≥ 2 s were considered astrocytic (Supplementary Video). Whole (x-y-time) Ca^2+^ events were identified in local astrocytic syncytium as previously described for single astrocytes (Figure 4B) (Wu et al., 2014). The frequency of Ca^2+^ events normalized to the imaged area (frequency density) was not significantly different between control and SE rats (frequency density: 5.3 ± 1.8 s^−1^ mm^−2^, *n* = 7, in control; 3.8 ± 0.8 s^−1^ mm^−2^, *n* = 7, after SE; *p* = 0.5, Mann-Whitney test, Figure 4C). Consistent with the previous report the distributions of event sizes (S_max_, maximal projection) and of events durations followed a power law (Wu et al., 2014):

(15) $$\begin{array}{l} P\left(x\right)\sim x^{-\alpha }, \end{array}$$

where *P*–probability, *x*–analyzed parameter (S_max_ or duration), α–power law exponent. α defines mean value and standard deviation of the sample. Smaller α suggests that distribution has higher proportion of larger or longer events, and vice versa. To calculate α the sample was log-binned and fitted with power function. The α of S_max_ was significantly larger after SE, suggesting a smaller proportion of large Ca^2+^ events in the distribution [α(S_max_): 2.7 ± 0.3, *n* = 7, in control; 3.5 ± 0.2, *n* = 7, after SE; *p* = 0.015, Mann-Whitney test, Figure 4D]. No significant difference in α of durations was observed after SE [α(durations): 2.75 ± 0.23, *n* = 7, in control; 3.02 ± 0.24, *n* = 7, after SE; *p* = 0.15, Mann-Whitney test, Figure 4E]. This finding suggests that morphological changes in astrocytes correlate with reduction of large-sized Ca^2+^ events in astrocytic syncytium after SE.

**Figure 5:**
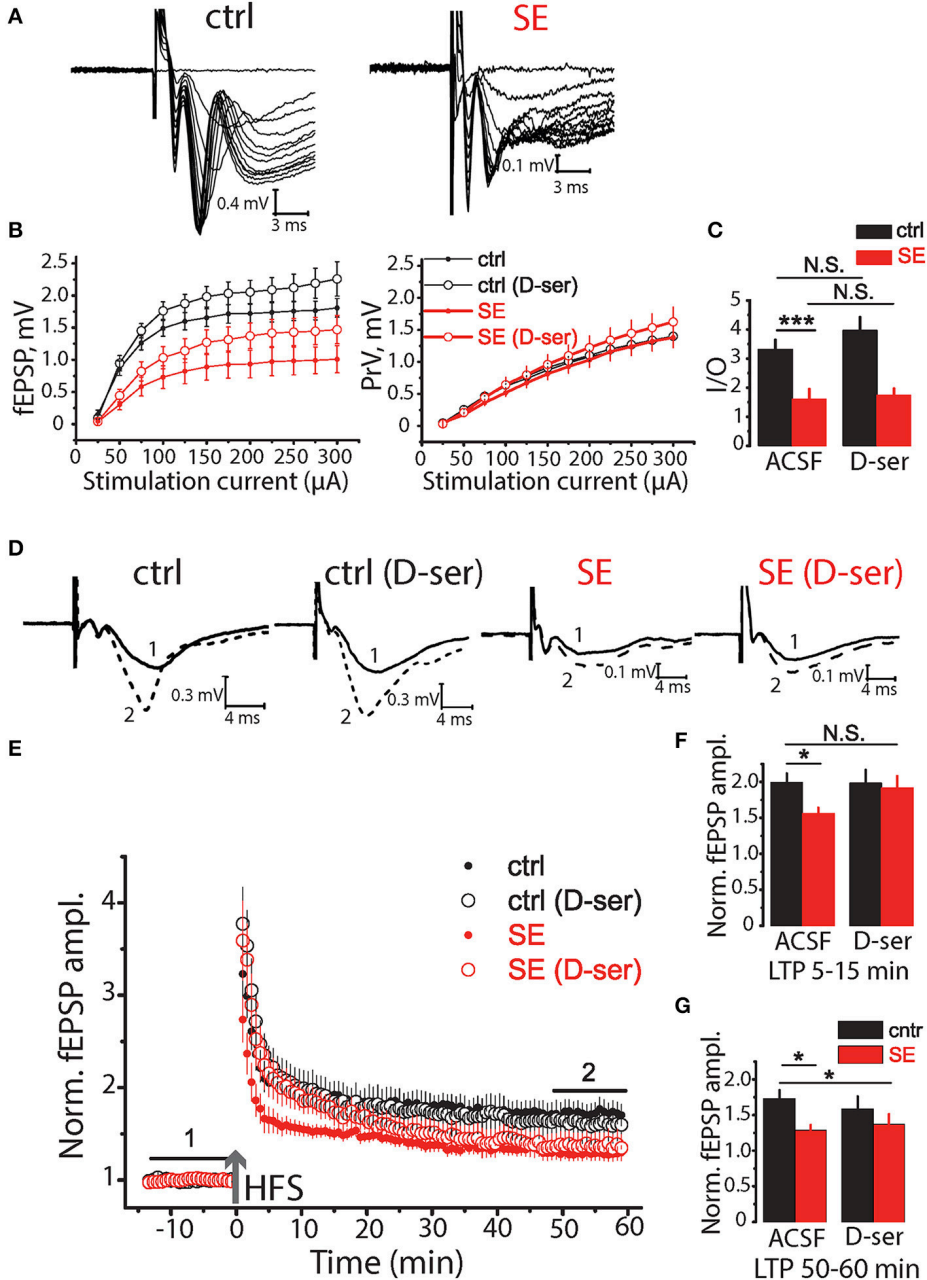
Effects of D-serine on synaptic neurotransmission in the hippocampus of control and SE-rats. **(A)** Representative examples of fEPSPs recorded in the hippocampal CA1 of control (ctrl) and SE-rats (SE). **(B)** Input-output (I/O) curves for the fEPSPs (*left*) and PrV (*right*). **(C)** Bar graphs showing the maximum slope of I/O curves for the fEPSPs. **(D)** Traces are the average of fEPSPs recorded during baseline (1) and 47–60 min after HFS (2). **(E)** LTP induced by HFS in the CA1 region of the hippocampus of control and SE-rats. Note that application of D-serine (D-ser) does not affect the magnitude of LTP in control rats but fully restored initial phase of LTP (5–15 min) in SE-rats. **(F,G)** The summary bar graphs showing significant reductions in LTP after SE as measured by the average normalized fEPSP amplitude and the effect of D-serine on initial **(F)** and later **(G)** phase of LTP. The bars with error bars are means ± SEMs. ***p < 0.01 *p < 0.05, N.S. *p* > 0.05 t-test.

Ca^2+^ activity in astrocytes is responsible for a number of functions including the release of gliotransmitters such as *D*-serine (Papouin et al., 2017). *D*-serine acts as a co-agonist of NMDA receptors and is required for long-term potentiation (LTP) (Henneberger et al., 2010, 2012). Thus, the decrease of astrocytic Ca^2+^ activity can reduce the amount of *D*-serine released and thus impair LTP after SE (Sherwood et al., 2017). To test this hypothesis, we recorded field (f)EPSPs in CA1 *str.radiatum* in response to stimulation of Shaffer collaterals (Figure 5A) The relationship between fEPSP amplitude and stimulus strength has significantly decreased after SE [*F*_(11, 297)_ = 4.39, *p* < 0.001, two-way ANOVA; Figures 5B,C], but no significant difference in relationship between presynaptic fiber volley (PrV) and stimulus strength was observed [*F*_(11, 275)_ = 0.37, *p* = 0.97, two-way ANOVA; Figures 5B,C]. The maximum rise slope of the input-output (I/O) relationships (fEPSP amplitude vs. PrV amplitude) was lower after SE (control: 3.32 ± 0.32, *n* = 13, SE: 1.61 ± 0.33, *n* = 14, *p* < 0.01, *t*-test; Figure 5C). These results suggest that the number of fibers or excitability of Shaffer collaterals did not change (unless the decreased number of fibers is compensated by their higher excitability), but the number of activated synapses decreased after SE. This result is consistent with neurodegeneration observed after SE. 10 μM *D*-serine changed the relationship between fEPSP amplitude vs. stimulus strength both in control [*F*_(11, 231)_ = 2.23, *p* < 0.05, two-way ANOVA; Figures 5B,C] and after SE [*F*_(11, 330)_ = 2.13, *p* < 0.05, two-way ANOVA; Figures 5B,C], however, *post hoc* LSD test did not confirm the effect of *D*-serine at any level of stimulation.

Three trains of high-frequency stimulation (HFS, 100 pulses at 100 Hz, with an inter-train interval of 20 s) were applied to induce LTP (Henneberger et al., 2010). The LTP magnitude was significantly lower after SE compared to control animals (1.73 ± 0.12, *n* = 14, in control 50–60 min after induction; 1.29 ± 0.07, *n* = 15, after SE; *p* < 0.01, *t*-test; Figures 5D–G). *D*-serine did not significantly affect the magnitude of LTP in control (*p* = 0.48, *t*-test; Figures 5D–G), but restored initial phase of LTP (5–15 min) after SE (1.92 ± 0.17, *n* = 13) to the level of control animals (1.99 ± 0.13, *n* = 14, *t*-test = 0.35, *p* = 0.73). LTP enhancement by exogenous *D*-serine in a later phase of LTP (50–60 min) was not however significant (1.37 ± 0.14, *n* = 13, *t*-test = 0.54, *p* = 0.60, Figure 5G).

Our findings suggest that neurodegeneration 2–4 weeks after pilocarpine-induced SE is accompanied by re-modeling of astrocytes and reduction of their coupling through gap-junctions. It does not affect the ability of astrocytes to clean up extracellular K^+^ despite the expected decrease in its spatial buffering. Decreased number of astrocytic branches correlates with reduced sizes of spontaneous Ca^2+^ events in astrocytes. This in turn may be responsible for *D*-serine deficiency and impaired LTP after SE.

## Discussion

Astrocytic processes often comprise of thicker astrocytic branches and thinner, nanoscale leaflets (Bernardinelli et al., 2014a; Khakh and Sofroniew, 2015). Electron microscopy studies have suggested that thicker astrocytic branches contain endoplasmic reticulum and mitochondria serving as endogenous Ca^2+^ stores (Reichenbach et al., 2010; Patrushev et al., 2013; Bernardinelli et al., 2014b). These structures are thought to be responsible for the amplification of Ca^2+^ signals and their propagation within the astrocyte. The leaflets are thin, sheet-like structures connected to branches. They have the minimal volume of cytoplasm and seem to be largely devoid of organelles. The high surface-to-volume ratio of leaflets ensures the high surface of their plasma membrane within the limited volume of brain tissue (Lehre and Rusakov, 2002). This makes leaflets highly efficient for neurotransmitter uptake and K^+^ clearance. Perisynaptic leaflets form an “astrocytic cradle” around synapses (Verkhratsky and Nedergaard, 2014). Like dendritic spines, astrocytic leaflets are highly plastic; morphological plasticity of perisynaptic leaflets regulates synaptic coverage (Witcher et al., 2007; Reichenbach et al., 2010; Bernardinelli et al., 2014a; Heller and Rusakov, 2015). For example, reduced IP_3_-dependent Ca^2+^ activity in astrocytes leads to reduced coverage of spines by astrocytic processes and enhanced glutamate spillover (Tanaka et al., 2013). Changes in the astrocytic environment are also reported following synaptic plasticity (Bernardinelli et al., 2014a).

Our morphological analysis revealed a striking difference between SE and control astrocytes. The Sholl analysis was performed on astrocytic processes identified with two-photon laser scanning microscopy (astrocytic branches ~>0.5 μm in diameter). Although this analysis did not reveal a difference between the samples in the enclosing radius (size of the astrocytic domain) and number of primary astrocytic branches, a significant reduction in the number of distal astrocytic branches was detected. However, astrocytic leaflets are very thin and are beyond the diffraction-limited light microscopy resolution. To assess possible changes in leaflets VF, we plotted a profile of leaflets fluorescence normalized to the fluorescence of soma (Medvedev et al., 2014; Heller and Rusakov, 2015). This method assumes that, with a ~1 μm thick two-photon excitation plane, somatic fluorescence originates from the dye that occupies ~100% of the visualized volume whereas fluorescence of the leaflets area is determined by tissue volume fraction occupied by the cytoplasm of all leaflets in the excitation region. Thus, the ratio between the non-saturated soma fluorescence and the fluorescence of leaflet area is roughly proportional to the VF of leaflets. No significant difference in this ratio was found between astrocytes of SE and control animals. Nevertheless, equal VFs of leaflets do not rule out nanoscopic redistribution of leaflets that affect synaptic coverage.

To get further insights into the spatial organization of astrocytic processes, we advanced a novel, spatial complexity-entropy spectra analysis. This analysis evaluates the nature of order and structure prevalence in a system. Entropy monotonically increases as the system goes from a highly ordered crystalline-like structure to a completely disordered state lacking internal structure. Statistical complexity, on the other hand, changes non-monotonically and reaches a maximum at intermediate levels of entropy, falling near zero for the two extremes of orderliness in a system. Intuitively, the complexity of regular patterns is low due to its predictability and the complexity of a disordered state is low due to the simplicity of its statistical description. Between the two extremes, a given entropy level can correspond to a range of complexity levels. Systems near critical states or in dynamical chaos regimes are characterized by high levels of complexity (Rosso et al., 2007). In such systems, disorder is non-trivial, and the systems contain both structure and randomness. Branching patterns of astrocytic processes are an example of such a non-trivial structure, where large astrocytic branches correspond to locally ordered structures, whereas fine ramifications of leaflets are seen as more complex and chaotic. Thus, entropy-complexity spectra will reflect the ratio between astrocytic branches and leaflets. Consistent with other morphological analysis we observed a shift in entropy-complexity spectra of astrocytes corresponding to a decrease in branches/leaflets ratio.

Astrocytic coupling through gap junctions has been suggested as a mechanism for K^+^ “spatial buffering” in the central nervous system (Kofuji and Newman, 2004; Meeks and Mennerick, 2007). K^+^ released during action potential propagation and during activation of postsynaptic glutamate receptors is removed by astrocytes through various mechanisms (Walz, 2000; Dallérac et al., 2013; Shih et al., 2013; Pekny et al., 2016; Lebedeva et al., 2018). Once taken by astrocytes, K^+^ quickly re-equilibrates within an astrocytic syncytium through gap-junctions which are permeable to this ion. One counterargument to this hypothesis is that intracellular K^+^ concentration does not considerably change due to the removal of K^+^ from narrow intercellular cleft to the large volume of cytoplasm. To test if astrocytic remodeling and uncoupling through gap-junctions affect K^+^ clearance, we recorded I_K_ in astrocytes in response to synaptic stimulation. No significant difference in the amplitude and the timecourse of I_K_ was detected between SE and control animals. Thus, in conditions of synchronous synaptic activity, K^+^ clearance did not change after SE. It rasises the question whether gap junction suppression could contribute significantly for excessive K^+^ accumulation in epileptogenesis.

Astrocytic remodeling may occur as a result of neurodegeneration which is pronounced 2 weeks after SE (Curia et al., 2008). Fewer neurons make fewer synaptic connections which need less astrocytic processes to support them. Reduction in a number of distal astrocytic branches and gap-junction coupling may also decrease Ca^2+^ events spread in single astrocytes and astrocytes syncytium. Spreading of Ca^2+^ events depends on Ca^2+^ release from endogenous stores. When some of the distal astrocytic branches containing Ca^2+^ stores are abolished, Ca^2+^ transients starting in leaflets have less chance to be amplified after SE. Astrocytic gap-junction may play a role in the propagation of Ca^2+^ signals in astrocytic syncytium (Enkvist and Mccarthy, 1992; Höfer et al., 2002; Fujii et al., 2017). When astrocytes become uncoupled, Ca^2+^ events spread may be limited. This, however, does not exclude a possibility of Ca^2+^ activity spread by the release of gliotransmitters (e.g., ATP) (Guthrie et al., 1999; Cotrina et al., 2000). Overall, we found that morphological changes correlated with the reduced spread of Ca^2+^ events in astrocytic syncytium observed after SE. Diminished Ca^2+^ signaling in astrocytes may affect several astrocytic functions such as release of gliotransmitters (Zorec et al., 2012; Araque et al., 2014), Ca^2+^ dependent K^+^ clearance (Wang et al., 2012) or outgrow of perisynaptic astrocytic processes (Tanaka et al., 2013; Heller and Rusakov, 2015). This can not only affect signaling in astrocytic syncytium, but also neuronal excitability, activation of extrasynaptic receptors, synaptic transmission, and plasticity (Rusakov et al., 2011; Zorec et al., 2012; Verkhratsky and Nedergaard, 2018).

In the present study, we have demonstrated hippocampal synaptic dysfunctions in pilocarpine-treated rats, as assessed by the reductions in both basal transmission and LTP at Schaffer collateral-CA1 synapses. The decrease in basal transmission is consistent with a reduction in the number of principal neurons in the CA3 hippocampal area of pilocarpine-treated animals observed earlier by us and other investigators in different hippocampal areas (see for review Curia et al., 2014). LTP induction at Schaffer collateral-CA1 synapses is an NMDA receptor-dependent form of synaptic plasticity (Malenka and Bear, 2004) and, therefore, the impairment of LTP most likely indicates the disturbance in NMDA receptor signaling. NMDA receptor signaling might be disturbed in many ways in epilepsy. For example, it might be affected because of the perturbations in the glutamate-glutamine cycle, such as increased extracellular levels of glutamate, loss of astrocytic glutamine synthetase, and changes in glutaminase and glutamate dehydrogenase, which are frequently encountered in patients with epilepsy (Coulter and Eid, 2012; Eid et al., 2016). Another possible reason is the changes in the production of individual subunits of NMDA receptors, which have been shown in several experimental models of epilepsy including the lithium-pilocarpine model (Di Maio et al., 2013; Müller et al., 2013; Peng et al., 2016; Amakhin et al., 2017). Hippocampal astrocytes in brain slices retain the ability to control LTP within or near their individual territories involving Ca^2+^-dependent *D*-serine release (Henneberger et al., 2010). This implies that astrocytes are at least capable of regulating local *D*-serine supply and might indeed be able to deliver *D*-serine to specific NMDA receptor populations. Therefore, our next set of experiments was designed to evaluate the role of *D*-serine signaling. We found that application of *D*-serine fully restored the initial phase of LTP (5–15 min) after SE.

Morphological and functional integrity of astrocytes is a key to the healthy brain. Astrocytes occupy non-overlapping or slightly overlapping spatial domains (Bushong et al., 2002) which they fill with highly ramified processes (Khakh and Sofroniew, 2015). Such architecture helps to support neurons metabolically, maintain local homeostasis, synaptic plasticity etc. (Verkhratsky and Nedergaard, 2018). Disruption of the astrocytic domain organization, morphological alterations of astrocytes and changes in the number of these cells are characteristic to many of brain disorders (Verkhratsky et al., 2017). It appears that the changes in astrocytes often precede neurodegeneration and clinical symptoms (Rossi and Volterra, 2009). Astrocytic pathologies can be of several types: (1) changes in the number of astrocytes, e.g., astrogliosis or astrodegeneration; (2) astrocytic remodeling, e.g., atrophy; and (3) reactive astrogliosis. A combination of such astrocytic pathologies might signify of a particular disease or of a stage of the disease (Verkhratsky and Parpura, 2016). Astrocyte degeneration and atrophy have been described in the hSOD1 mouse, an experimental model of amyotrophic lateral sclerosis (Rossi et al., 2008). Astrocytic markers are reduced in the Parkinson's disease (Tong et al., 2015). Region- and stage-specific alterations in astrocytic morphology have been reported in the Alzheimer's disease (Olabarria et al., 2010; Rodríguez-Arellano et al., 2016). Loss of astrocytic domain organization has been observed in the epileptic brain (Oberheim et al., 2008). Here we find that the lithium-pilocarpine model of SE is associated with the atrophy of distal astrocytic branches and with astrocyte uncoupling reported earlier (Bedner et al., 2015). This, in turn, diminishes Ca^2+^ activity in these cells and correlates with the *D*-serine dependent impairment of LTP initiation. However, the LTP maintenance phase failure was not related to the insufficient *D*-serine supply. The maintenance phase could be potentially linked to reduced supply of energy substrates from atrophic astrocytes or to a malfunction of any other metabolic or homeostatic support of synapses. However, this hypothesis requires further experimental testing. Our results demonstrate that the astrocyte remodeling after SE does not affect VF of astrocytic leaflets and K^+^ clearance. This indicates that astrocytes can effectively maintain synaptic microenvironment at moderate levels of synaptic activity. However, if astrocytes can prevent the K^+^ accumulation during synchronized epileptiform activity remains unclear. In agreement with previous reports we observe astrocyte uncoupling through the gap-junctions (Bedner et al., 2015). This may affect spatial buffering of excessive amounts of K^+^ that are released during focal seizures (Fröhlich et al., 2008). In addition, optical methods, which we used, assess the synaptic microenvironment very indirectly. More careful studies on the integrity of “astrocytic cradle” around synapses are required with the use of super-resolution light microscopy or electron microscopy.

## Ethics statement

All the animal experiments were approved by N. I. Lobachevsky State University of Nizhny Novgorod ethics committee.

## Author contributions

APl, AL, PD, ON, and TP performed the experiments, analyzed the data, prepared the figures. AP wrote the MATLAB script for Ca^2+^ analysis. AB wrote the scripts for Sholl analysis and entropy-complexity analysis, analyzed the data, prepared the figures. AZ analyzed the data, prepared the figures. DR planned the experiments. AS planned the experiments, supervised the project. AB, AZ, DR, and AS wrote the paper.

### Conflict of interest statement

The authors declare that the research was conducted in the absence of any commercial or financial relationships that could be construed as a potential conflict of interest.
