# Supplementary material for: Astrocytic Atrophy Following Status Epilepticus Parallels Reduced Ca2+ Activity and Impaired Synaptic Plasticity in the Rat Hippocampus
Source: Front Mol Neurosci. 2018 Jun 26;11:215. doi: 10.3389/fnmol.2018.00215 (PMC6028739; doi:10.3389/fnmol.2018.00215)
Supplement: Supplementary file 2 [file Data_Sheet_1.pdf]

Astrocytic atrophy following *status epilepticus* parallels reduced Ca^{2+} activity and impaired synaptic plasticity in the rat hippocampus

Alex Plata, Albina Lebedeva, Pavel Denisov, Olga Nosova, Tatiana Y. Postnikova, Alexey Pimashkin, Alexey Brazhe, Aleksey V. Zaitsev, Dmitri A. Rusakov, Alexey Semyanov

Supplementary figures

Supplementary figure 1 Lithium-pilocarpine model of SE

The development of SE in animals, that was identified according to the Racine scale with the following stages: 0 - no abnormality; 1 - mouth and facial movements; 2 - head nodding; 3 - forelimb clonus; 4 - rearing; 5 - rearing and falling (Phelan et al., 2015). **A.** The images of rats expressing $4^{th}/5^{th}$ stages of seizures. If the generalized seizures lasted for 20 min, we considered it as SE. **B.** Summary data sowing the mean number of episodes of different types of seizures developed by rats after pilocarpine injection.

Supplementary Fig.2 Preparation of images for Sholl analysis

A. Preprocessing. Z-stacks were re-sampled to the same lateral resolution of 0.25 µm/px. The following filtering procedure was applied to each Z-plane independently: (1) high-pass filtering by taking a difference between a Gauss-smoothed frame with $\sigma = 0.5$ and a Gauss-smoothed frame with $\sigma = 32$; (2) coherence-enhancing diffusion filtering (CEDF) (Weickert and Scharr, 2002)with stopping time T = 50. This kind of nonlinear image processing procedure allows to recover and enhance filament-like like structures. The filtered Z-stacks were then collapsed to 2D images by max-projection along the Z-axis. The combination of high-pass filter and CEDF provided much more detailed projections in comparison to max-projections of raw stacks or after application of the high-pass filter alone (B).

B. Thresholding. The projected images were segmented into regions containing astrocytic processes (foreground) and background. First, the images were adaptively thresholded by labeling as foreground such pixels, where intensity value was larger than the average over a 3x3 sliding window. This labeling was excessive, and only contiguous structures containing more than 100 pixels were retained for the next steps. The resulting binary image was again filtered with CEDF (T = 50) to promote filament-like strings of foreground pixels and thresholded at intensity level 0.5. The final mask used for Sholl analysis was obtained by removing all structures containing less than 100 pixels.

C. An example overlay of the projected image, filtered image and a process mask

Supplementary Fig.3 Sholl analysis parameters

Except for remodeling of distal branches other parameters of astrocytes in SE assessed with Sholl analysis appeared unchanged. **A.** First intersections define the number of primary astrocytic branches originating from soma. **B.** Max. intersections define the maximal number of resolved astrocytic branched. **C.** Enclosing radius – the size of astrocytic domain. **D.** Max. inter. radius – the distance from the center of astrocytes where the number or resolved branches is highest.

References

Phelan, K.D., Shwe, U.T., Williams, D.K., Greenfield, L.J., and Zheng, F. (2015). Pilocarpine-induced status epilepticus in mice: A comparison of spectral analysis of electroencephalogram and behavioral grading using the Racine scale. *Epilepsy Res* 117**,** 90-96.

Weickert, J., and Scharr, H. (2002). A Scheme for Coherence-Enhancing Diffusion Filtering with Optimized Rotation Invariance. *Journal of Visual Communication and Image Representation* 13**,** 103-118.

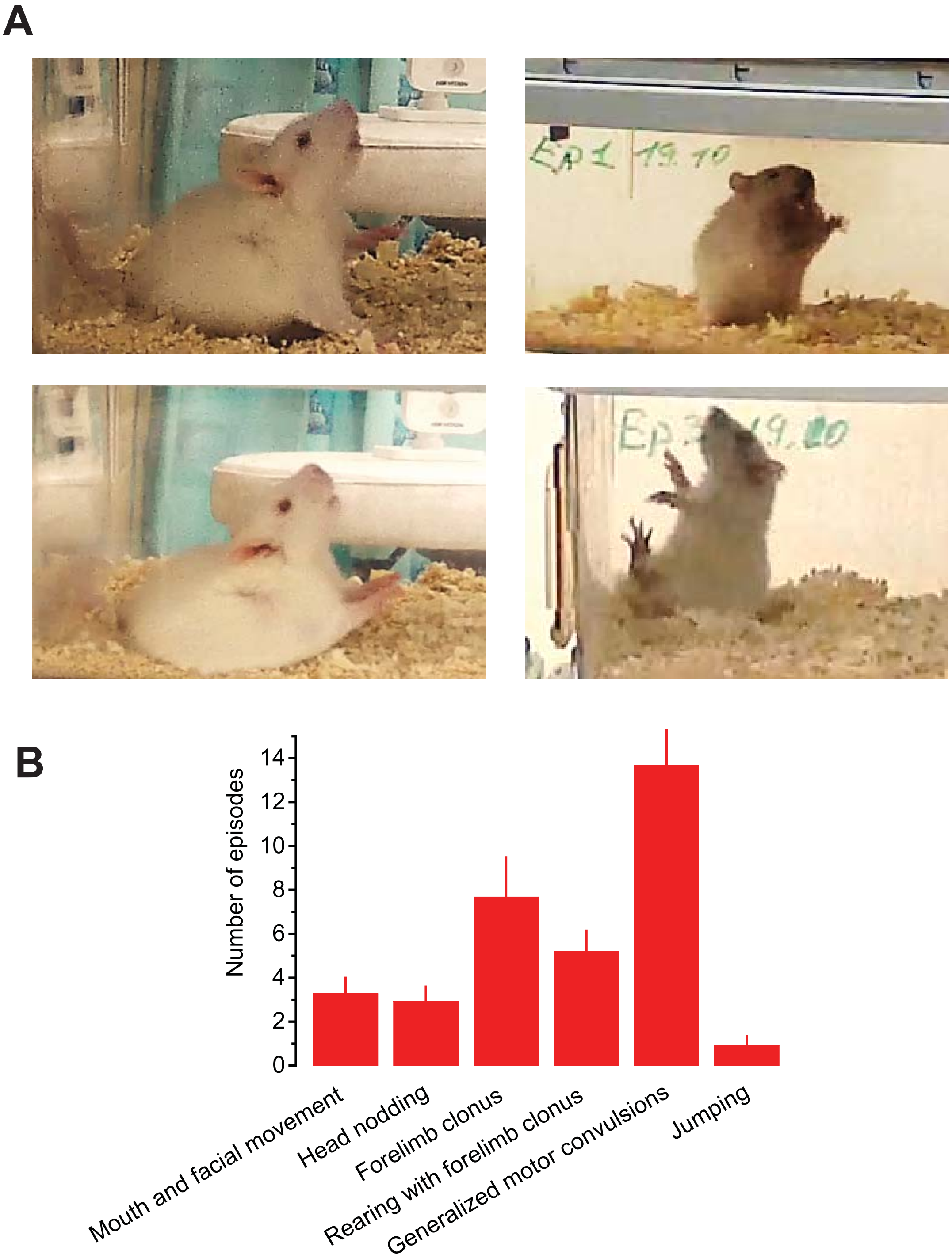

Supplementary Figure 1

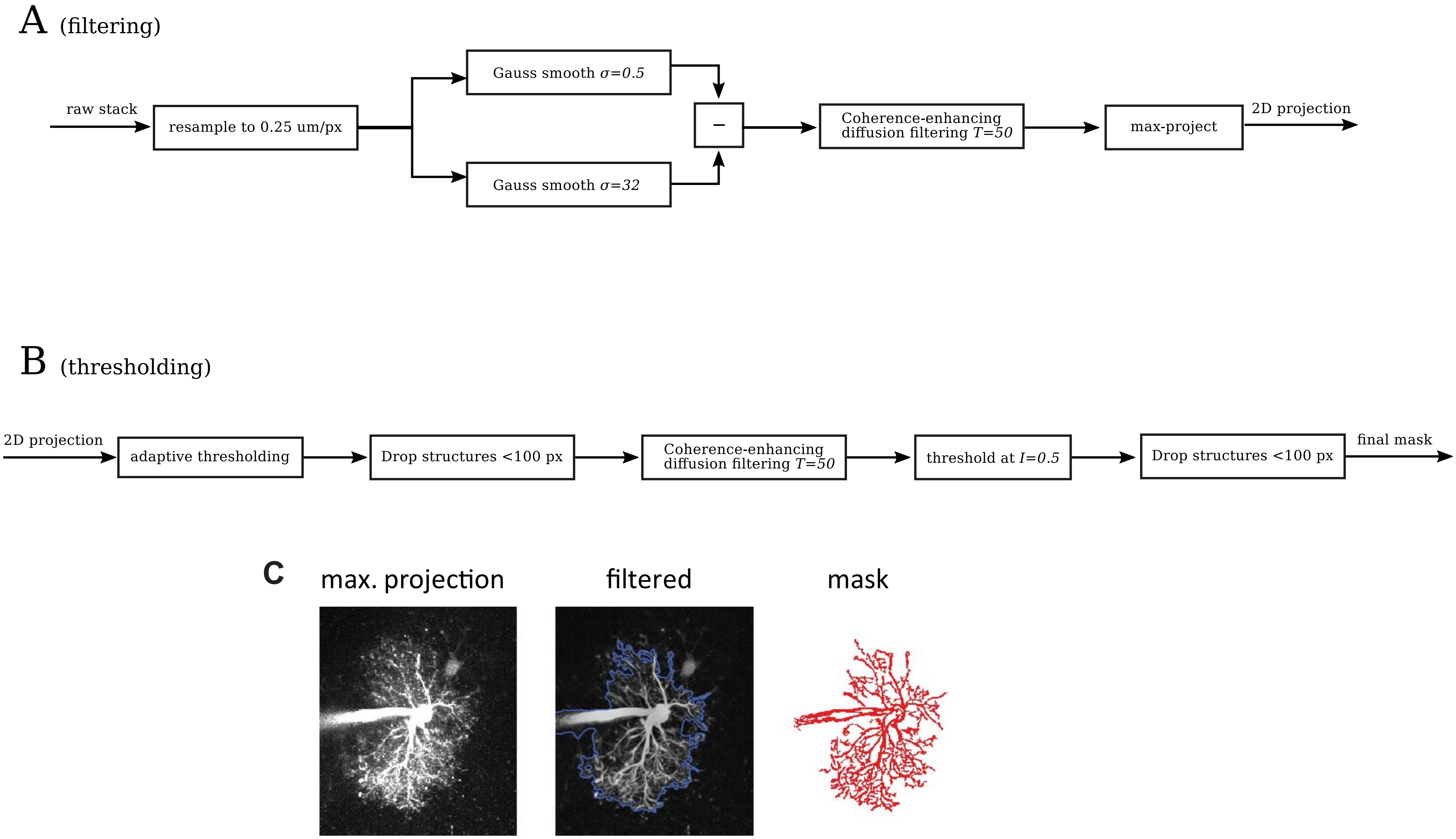

Supplementary Figure 2

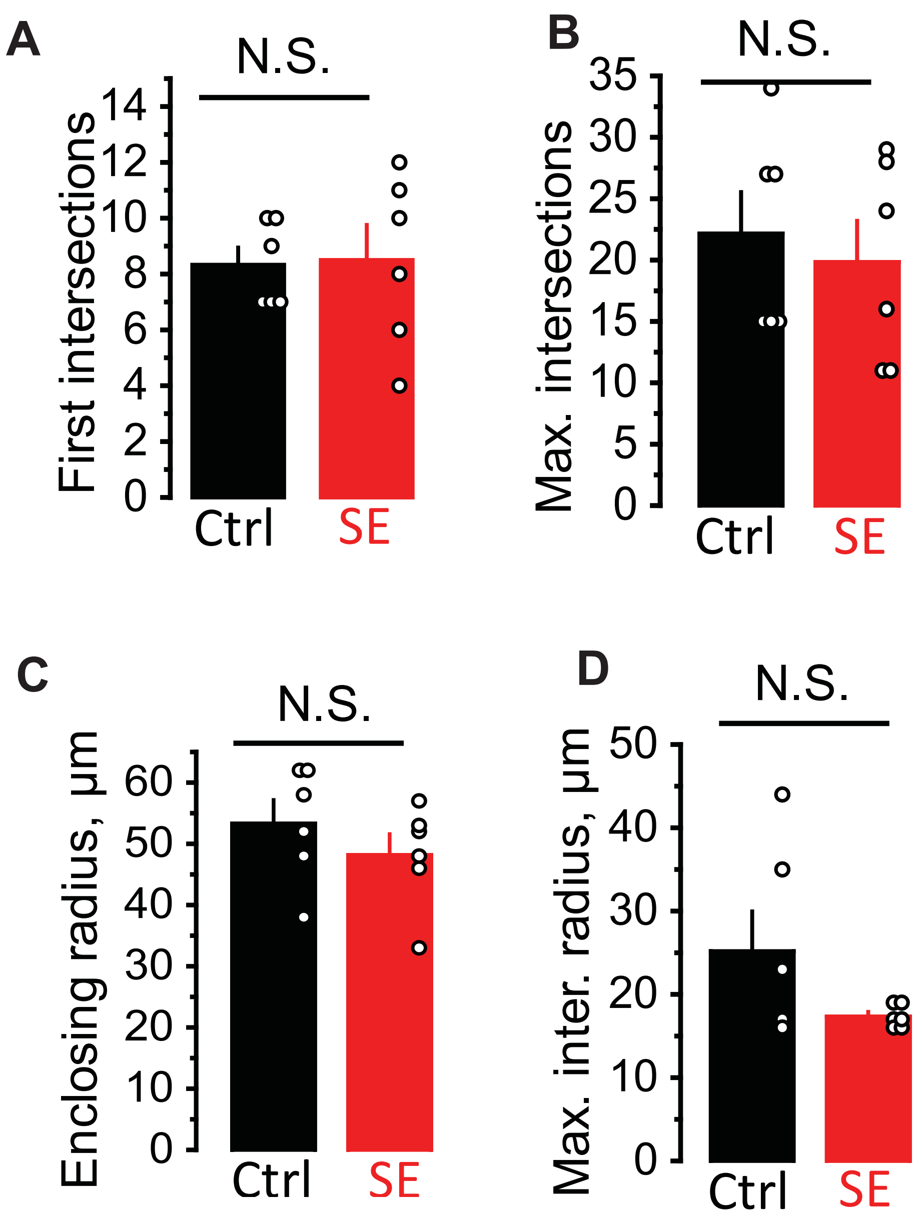

Supplementary Figure 3
